# Heat Shock Factor 1 as a Prognostic and Diagnostic Biomarker of Gastric Cancer

**DOI:** 10.3390/biomedicines9060586

**Published:** 2021-05-21

**Authors:** Woong Kim, Seok-Jun Kim

**Affiliations:** 1 Department of Integrative Biological Sciences & BK21 FOUR Educational Research Group for Age-Associated Disorder Control Technology, Chosun University, Gwangju 61452, Korea; kw@chosun.ac.kr; 2 Department of Biomedical Science, College of Natural Sciences, Chosun University, Gwangju 61452, Korea

**Keywords:** gastric cancer, heat shock factor 1 (HSF1), diagnosis, prognosis, biomarker

## Abstract

Identification of effective prognostic and diagnostic biomarkers is needed to improve the diagnosis and treatment of gastric cancer. Early detection of gastric cancer through diagnostic markers can help establish effective treatments. Heat shock factor 1 (HSF1), presented in this review, is known to be regulated by a broad range of transcription factors, including those characterized in various malignant tumors, including gastric cancer. Particularly, it has been demonstrated that HSF1 regulation in various cancers is correlated with different processes, such as cell death, proliferation, and metastasis. Due to the effect of HSF1 on the initiation, development, and progression of various tumors, it is considered as an important gene for understanding and treating tumors. Additionally, HSF1 exhibits high expression in various cancers, and its high expression adversely affects the prognosis of various cancer patients, thereby suggesting that it can be used as a novel, predictive, prognostic, and diagnostic biomarker for gastric cancer. In this review, we discuss the literature accumulated in recent years, which suggests that there is a correlation between the expression of HSF1 and prognosis of gastric cancer patients through public data. Consequently, this evidence also indicates that HSF1 can be established as a powerful biomarker for the prognosis and diagnosis of gastric cancer.

## 1. Introduction

According to the GLOBOCAN 2018 database, gastric cancer is considered the third leading cause of cancer-related deaths worldwide, following lung and colorectal cancer, in overall mortality [1]. Moreover, over a million new patients with gastric cancer are diagnosed every year [2]. Interestingly, the incidence of gastric cancer has steadily decreased over the past 50 years. This decline can be attributed to the rapid development of novel therapeutic techniques. Notably, the survival rates with gastric cancer have constantly improved due to early detection and successful treatment [1,3]. Moreover, due to early diagnosis and treatment of gastric cancer, the 5-year survival rate is approximately over 90% [4]. Therefore, it is important and necessary to identify effective prognostic and diagnostic biomarkers and therapeutic targets of gastric cancer. However, in the case of gastric cancer, further research on the molecular biomarker is still needed because the understanding of molecular diagnostic and prognostic biomarkers remains poor [5,6,7,8]. Biomarkers are used as indicators of normal or abnormal states [9,10,11]. Despite the advances in research on cancer biomarkers, many biomarkers still lack detection, specificity, and sensitivity in gastric cancer [12]. Biomarkers can be classified based on the purpose of their use. Diagnostic biomarkers are used for early detection of disease [13,14]. Prognostic biomarkers provide information regarding the clinical or biological characteristics of the patients [13,15].

Heat shock factor 1 (HSF1) is an evolutionarily and indispensable component of transcription factor complex that maintains proteome homeostasis (proteostasis) in cells [16,17,18]. The cellular proteome homeostasis is vital to regulating protein synthesis, folding, maintenance, and degradation [19,20,21]. If various cellular or environmental cues that induce stress are stimulated in cells, the state of proteome homeostasis is disrupted [19]. In stress conditions, HSF1 acts as a key regulator that induces transcription of heat shock proteins (HSPs) [16,22]. HSPs induce the expression of chaperone genes and control cellular proteome homeostasis, such as folding of proteins and refolding of misfolded proteins [19,23,24,25]. Similarly, HSF1 can mediate protein stability through maintaining cell functionality and viability during stress [26]. In addition, numerous reports demonstrated that the expression of HSF1 was notably elevated in various human cancers [27,28,29,30,31,32,33,34,35]. In cancer cells, protein synthesis is elevated upon increased ribosomal biogenesis [36,37]. HSF1 transcriptional activity is closely related to protein translation, and thus, it mediates protein synthesis and induces remodeling to support the tumor state [38]. Moreover, HSF1 is not only involved in proteome homeostasis but also in cell proliferation, invasion, migration, and metabolism [29,31,39,40]. Recently, studies on the molecular mechanism of HSF1 have been conducted in gastric cancer [41,42,43]. In gastric cancer cells, the presence of HSF1 promotes the effect of tumorigenesis. Additionally, it is one of the most studied potential molecular biomarkers of gastric cancer. In this review, we summarize the important studies on the activation and role of HSF1 in gastric cancer. Moreover, we suggest a role of HSF1 as a potential biomarker of gastric cancer.

## 2. Structure and Function of HSF1

### 2.1. The Discovery of HSF1 and Its Structure

Heat shock response was initially discovered in 1962 [44], and HSF1 and various other HSFs have also been discovered [45,46,47]. The molecular structure of HSF1 is highly conserved from yeast to mammals. HSF1 consists of an N-terminal, helix-turn-helix DNA-binding domain (DBD), and an oligomerization domain that comprises hydrophobic heptad repeats (HR-A/B) [48,49]. Oligomerization of HSF1 is mediated by the interaction of coiled-coil and HR-A/B domains, and consequently, HSF1 binds to DNA via DBD as a trimer [50,51,52]. Since HSF1 possesses the inverted nGAAn pentamer sequences, the trimer recognizes a specific nGAAn sequence in DNA [53,54,55]. Spontaneous oligomerization of HSF1 is regulated by the C-terminal heptad repeat domain (HR-C). The HR-C domain regulates the trimerization of HSF1 through suppressing the intracellular interaction with the HR-A/B domain [51]. Additionally, HSF1 possesses a transactivation domain (TAD) located between the HR-A/B and HR-C domains. TAD is targeted by several proteins to regulate the extent of HSF1 activation and direct HSF1 to the specific target genes [56,57].

### 2.2. HSF1 in Cancers

In cancer, various environmental stressors, such as genotoxic, oxidative, inflammatory, and metabolic, induce tumor progression [58,59,60,61]. Under these stress conditions, HSF1 is elevated and activated, and thus, high HSF1 expression and activation is observed in various cancers. Moreover, cancer cells are easily mutated and exhibit high expression and activity of HSF1 [62]. Accumulating studies have demonstrated that the activation of HSF1 in cancers facilitates oncogenesis. The studies conducted by Dai and Min revealed that the silencing of HSF1 in mice that are deficient in p53 protected them from tumorigenesis [63,64]. Subsequently, the presence of HSF1 has been shown to induce tumor growth, invasion, and metastasis in melanoma and hepatocellular carcinoma (HCC) [39,65,66]. Conversely, the overexpression of HSF1 promotes tumor growth and metastasis in cooperation with the RAS/MAP kinase pathway [65,67]. Moreover, a relation between HSF1 expression and translation has also been recognized. HSF1 plays a role in stabilizing cancer proteome, and its silencing in cancer cells suppresses oncogenic proteins [68,69,70]. HSF1 facilitates the expression of HSPs, and it also supports tumorigenesis and tumor progression [27]. Interestingly, HSF1 deficiency has been shown to suppress the expression of HSPs, thereby mediating the inhibition of crucial signaling molecules and tumorigenesis [39]. Additionally, HSF1 silencing regulates ribosomal proteins, RPL13 and RPL17 [67]. These effects of HSF1 are associated with prognostic markers in various cancers [35]. These findings suggest that HSF1 induces tumorigenesis through the activation of growth, invasion, and metastasis, and it also mediates protein synthesis and stabilization of oncogenic proteins.

## 3. Role of HSF1 in Gastric Cancer

As mentioned earlier, the accumulating evidence in cancers for HSF1 activation suggests that it acts as a potent carcinogen in various cancers [22,26,71,72,73]. The role of HSF1 in these cancers is the same as in gastric cancer, which is tumor progression, including cell proliferation, invasion, and metastasis [41]. In the next section, we describe the biological role of HSF1 in the development of gastric cancer.

### 3.1. Proliferation and Apoptosis

It has been confirmed that HSF1 is highly expressed in various rapidly proliferating carcinomas [16,27,28]. This phenomenon also suggests that HSF1 is associated with a variety of biological functions in promoting tumor progression. Particularly, high expression of HSF1 exerts the same effect in the proliferation of gastric cancer cells [41,43]. Conversely, it has also been shown that HSF1 silencing inhibits the proliferation of gastric cancer cells [41,43]. The cell cycle is very important in regulating cell proliferation; therefore, we searched for reports on HSF1 and cell cycle in the literature. When S216 of HSF1 is phosphorylated, it was confirmed that HSF1 was degraded and promoted cell cycle arrest [74]. As a result, it was confirmed that the expression of HSF1 is associated with cell cycle regulation, as well as the resulting cell proliferation.

Additionally, under stress, HSF1 plays a fundamental role in improving cell survival through inhibiting apoptosis and cell death [71]. A study conducted by Aziz showed that HSF1, which was inhibited in gastric cancer cells, induced apoptosis through activating apoptosis-related proteins, such as Bax, caspase-3, -8, -9, and PARP [42]. HSP maintains not only the functional configuration and stability of cellular proteins but also the role of tumor-related proteins. HSF1 has been reported to act as a transcription factor that regulates the HSP genes [16,22]. A study conducted by Arora demonstrated that the HSP70 gene plays an important role in cell death in gastric cancer [75]. Additionally, it has been reported in several carcinomas that HSF1 regulates HSP70 [76,77]. As a result, it was confirmed that HSF1 regulates genes related to apoptosis and the expression of HSPs to modulate cell proliferation and cell death.

### 3.2. Invasion and Metastasis

In cancer patients, the spreading of cancer from the tissue of origin, and its continued growth in other organs, is a major life-threatening factor [78]. Therefore, the investigations of invasion and metastasis are important in cancer research.

Many studies indicated that cell motility for invasion and migration is promoted upon high expression of HSF1 in various cancer cells, such as breast cancer, hepatocellular cancer, and ovarian cancer [29,66,79]. Following this, Kim and Tong suggested that the presence of HSF1 promoted the invasion and migration activities in gastric cancer cells [41,43]. Among the evidence related to cytoskeleton and cell motility, the evidence that HSF1 regulates cell invasion and migration in gastric cancer suggests that HSF1 binds to the ArgBP2 promoter containing the sequence nGAAn [43]. The interaction of HSF1 with MORC2 mediates the invasion and migration of gastric cancer cells through inhibiting ArgBP2, which is an important regulator of cytoskeleton and cellular motility [43]. According to these findings, the presence of HSF1 mediates cell motility, thereby effecting invasion and migration in gastric cancer cells, which needs to be explored further in vivo.

### 3.3. Others

HSF1 plays a more diverse role in gastric cancer. A study conducted by Grunberg suggested that HSF1 upregulated inhibin subunit beta A (INHBA) and thrombospondin 2 (THBS2), which are involved in tumor progression [80]. INHBA and THBS2 from cancer-associated fibroblasts are packaged into extracellular vesicles and secreted into the tumor microenvironment to promote gastric cancer [80]. Abnormal regulation of metabolism affects the development and progression of cancer cells. In chemo-resistant gastric cancer cells, HSF1 promotes the transcription of pyruvate dehydrogenase kinase 3 (PDK3). Thereafter, PDK3 stimulates glycolysis through inhibiting pyruvate dehydrogenase (PDH). Furthermore, PDK3 prevents ubiquitination-dependent degradation of HSF1. Therefore, HSF1 and PDK3 promote glycolysis and chemoresistance in gastric cancer through a positive feedback loop [81]. *Helicobacter pylori* (*H. pylori*) is a gastric bacterial pathogen, and its infection is a major risk factor associated with gastric cancer [82,83,84]. Cytotoxin-associated gene A (CagA) is a virulence factor of *H. pylori*, and it plays an important role in the development of gastric cancer [85]. Gastric cancer cells treated with *H. pylori* CagA inhibited apoptosis through stimulating FUT4 fucosylation, which activated HSF1 transcription [42]. Collectively, these findings reinforce the fact that HSF1 aptly orchestrates an extensive network of cellular functions to facilitate robust oncogenesis systemically.

## 4. Clinicopathological Characteristics of HSF1 in Gastric Cancer

### 4.1. HSF1 Expression in Gastric Cancer

HSF1 is aberrantly expressed in various cancers [27,28,67]. As previously mentioned, HSF1 is a transcription factor that is mainly expressed inside the cytoplasm and nucleus, but it is also linked to the extracellular secretion [16,17,18]. Therefore, HSF1 expression is assessed for tissue-specific detection in gastric cancer, but it exhibits the advantage of predicting the expression patterns of various genes associated with gastric cancer progression. The study conducted by Tong revealed that the gene expression of HSF1 was high in 21 out of 32 gastric cancer tissues [43]. Through the seven public GEO datasets, HSF1 in gastric cancer tissues exhibited high expression, which was more than that in normal tissue (Table 1). Additionally, TCGA data also revealed the overexpression of HSF1 in gastric cancer tissues [86]. Moreover, both gene and protein expression levels of HSF1 have also been reported to be elevated in tumor tissues of gastric patients [41,43,86]. Although HSF1 levels have not been compared at different cancer stages, they are shown to be elevated in gastric cancer patient tissues and are proposed to exhibit a potential role in diagnosing gastric cancer.

### 4.2. Impact of HSF1 on the Survival of Gastric Cancer Patients

**Figure 1:**
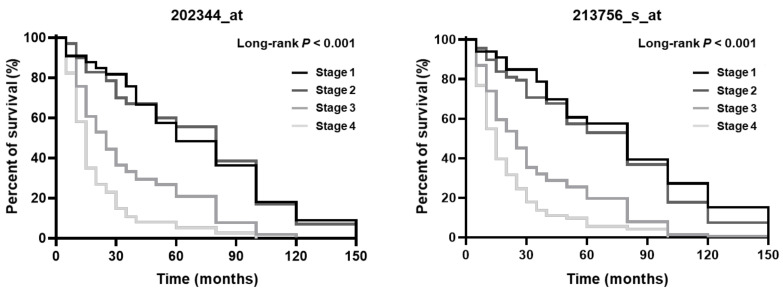
HSF1 expression was correlated with the overall survival of gastric cancer patients. Two probes (Affy ID: 202344_at, and Affy ID: 213756_s_at) revealed that high HSF1 expression leads to poor survival depending on gastric cancer patient stage. The Kaplan–Meier survival curves were generated using the KM-plotter online analysis tool.

High HSF1 expression has been associated with poor survival in cancer patients [87,88,89]. The silencing of HSF1 reduced the incidence of tumors and increased the effects on long-term survival in mice [63]. In advanced stages of other cancers, the expression and activation of HSF1 has been shown to be elevated [31,66,90]. HSF1 has been suggested to play a role as a potential prognostic biomarker in other cancers. The studies conducted by Kim, Dai, and Grunberg revealed that high HSF1 expression also contributes to poor survival in gastric cancer patients [41,80,86]. Therefore, we confirmed the overall survival of gastric cancer patients with HSF1 expression using Kmplot public data (https://kmplot.com/analysis/, accessed on 2 January 2021). HSF1 overexpression in early (I and II) and advanced (III and IV) stages was associated with poor survival [86]. Additionally, for the overall survival, high HSF1 expression levels in gastric cancer patients led to poor survival in advanced cancer stages (Affy ID: 202344_at and 213756_s_at) (Figure 1, Table 2 and Table 3). Therefore, high HSF1 levels are associated with poor survival and worse long-term survival in advanced gastric cancer stages.

## 5. HSF1 as a Biomarker in Gastric Cancer

### 5.1. HSF1 as a Therapeutic Target

As a therapeutic target of gastric cancer, it is important to study the transcription factors involved in tumorigenesis and tumor progression [71]. Recent studies have demonstrated the potential of HSF1 as a target for gastric cancer therapy through the inhibition of the transcriptional activity of HSF1. Rocaglamide A and rohinitib (analogs to RocA) were found to disrupt HSF1 binding to the target genes and act as HSF1 inhibitors [91]. The studies conducted by Yoon suggested that KRIBB11 inhibits the transcriptional activity of HSF1 through disrupting the HSF1-dependent binding of p-TEFb (positive transcription elongation factor b) and hsp70 promoter [92]. Triptolide is derived from *T. wilfordii*, and it inhibits the trimerization of HSF1 complexes that bind to the endogenous HSP70 promoter [93]. Ginsenoside Rg3, the major compound in ginseng, was found to induce apoptosis through FUT4 inhibition via SP1 and HSF1 transcription regulation in gastric cancer cells with *H. pylori* CagA [42]. The inhibitory activities of triptolide [94,95,96,97] and KRIBB11 and KNK437 [81] were also confirmed in gastric cancer cell lines. Although not yet identified in gastric cancer, many HSF1 mediated inhibitors are being studied. Cantharidin [81,98] and Cardenolide CL-43 [99] have been shown to inhibit HSF1 transcriptional activity in cancer cells. In addition, PW3405 [100] and Compound 1 [101] were discovered as inhibitors of HSF1 by inhibition of phosphorylation. Quercetin [102,103,104] and Fisetin [76] are flavonoids identified as suppressors of the HSF1 binding to HSE. Additional HSF1-mediated inhibitors are summarized in Table 4. HSF1 inhibitors in gastric cancer are still in a preclinical stage; however, this evidence suggests that HSF1 acts as a potent target for gastric cancer therapy.

### 5.2. HSF1 Expression Level as a Prognostic and Diagnostic Biomarker

Biomarkers can be used for risk assessment of cancer and also for assisting in cancer staging for initial therapy [105]. Prognostic markers provide the information regarding the onset of cancer and can help identify the cancer patients requiring treatment. In cancer patients, the diagnostic biomarker can delay cancer progression through clinical management and suitable preventive interventions. Recent studies presented the possibility of employing HSF1 as a prognostic and diagnostic biomarker of gastric cancer. In gastric cancer tissue, mRNA and/or protein expression levels of HSF1 are significantly higher than those in normal tissue. Furthermore, HSF1 promotes the proliferation, invasion, and migration of gastric cancer cells [41,43,86]. Additionally, the expression level of HSF1 is associated with advanced tumor progression in gastric cancer patients. Kaplan–Meier analysis in patients with gastric cancer revealed that the high expression levels of HSF1 were associated with poor prognosis [41]. Dai et al. analyzed the prognostic value of HSF1 expression in early and advanced gastric cancer using TNM classification. It was confirmed that gastric cancer patients with higher HSF1 expression had worse overall survival rates and recurrence-free survival than those with low expression in early and advanced stages [43]. Altogether, HSF1 is associated with tumor progression and poor prognosis in gastric cancer, and it can serve as a prognostic and diagnostic biomarker of gastric cancer.

## 6. Conclusions

**Figure 2:**
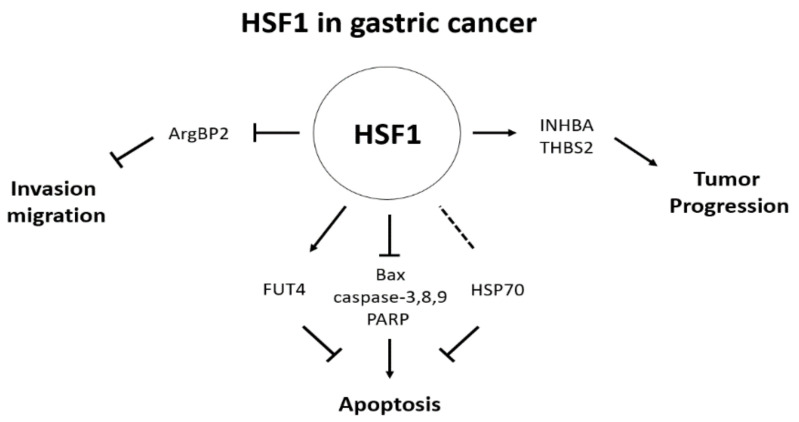
Various roles of HSF1 in gastric cancer. HSF1 has been shown to be involved in cell growth, apoptosis, migration, and invasion in gastric cancer.

As endoscopy and imaging technology developed for the diagnosis of gastric cancer, the detection of early gastric cancer-related lesions has also been improved; however, it is still important to identify additional biomarkers and study the mechanisms to assist in diagnosing gastric cancer at early stages. This review suggested that HSF1 can be employed as a diagnostic and prognostic biomarker of gastric cancer. Using several reports, we showed that HSF1 is a powerful multifaceted regulator of various cancers, including gastric cancer. Particularly, it was confirmed that the expression of HSF1 is increased in various cancers, including gastric cancer, and an increased HSF1 expression is involved in cell death through regulating genes (Bax, caspase-3, 8, 9, and PARP) related to apoptosis (Figure 2). It was confirmed that HSF1 exerts an anti-apoptotic effect. Additionally, it was confirmed that HSF1 regulates HSPs as a transcription factor of HSPs, thereby also affecting cell proliferation (Figure 2).

Additionally, HSF1 has been confirmed to exert an effect on cell migration and metastasis. Particularly, in gastric cancer, it has been reported to regulate cell migration through binding to ArgBP2, and HSF1 has been shown to regulate cell migration and metastasis (Figure 2). Moreover, it was confirmed that HSF1 is involved in the proliferation and metastasis of gastric cancer cells via an association with CagA, a major factor of *H. pylori*, which is known as a high-risk factor associated with gastric cancer (Figure 2). HSF1 expression has also been reported to be increased in various cancers as previously mentioned, and the increased expression of HSF1 in gastric cancer was confirmed through public data analysis (Table 1 and Figure 1). Based on these results, HSF1 has been suggested as an important regulator of gastric cancer cells, and its association with various signaling and target genes has been confirmed. These results confirm that HSF1 can be considered as an important biomarker for diagnosing gastric cancer and as a potential target for future drug development.

## Figures and Tables

**Table 1:** HSF1 expression profile in GEO datasets.

Datasets	Country	Year	Platform	Normal Tissue			Cancer Tissue			*p*-Value
				Number	Mean	SD	Number	Mean	SD	
GSE2685	Japan	2005	GPL80	8	7.57	0.83	22	8.20	0.24	0.0026
GSE13861	USA	2008	GPL6884	19	5.39	0.10	71	5.53	0.17	0.0017
GSE13911	Italy	2008	GPL570	31	7.40	1.16	38	8.82	0.91	<0.001
GSE29272	USA	2011	GPL96	134	5.68	0.41	134	5.84	0.53	0.0065
GSE54129	China	2014	GPL570	21	6.58	0.14	111	7.40	0.35	<0.001
GSE81948	Italy	2016	GPL6244	5	8.46	0.12	15	8.80	0.32	0.0368
GSE109476	China	2018	GPL24530	5	13.57	0.58	5	14.34	0.40	0.0398

**Table 2:** Overall survival of gastric cancer patients with high HSF1 expression according to the diseases stage (HSF1 probe: **Affy ID: 202344_at**).

Month (m)	Tumor Stage							
	Stage I		Stage II		Stage III		Stage IV	
	Number of Patients (%)		Number of Patients (%)		Number of Patients (%)		Number of Patients (%)	
0	33	(100.0)	70	(100.0)	153	(100.0)	74	(100.0)
5	30	(90.9)	68	(97.1)	139	(90.8)	61	(82.4)
10	30	(90.9)	63	(90.0)	116	(75.8)	43	(58.1)
20	28	(84.8)	58	(82.6)	81	(52.9)	20	(27.0)
40	22	(66.7)	47	(67.1)	45	(29.4)	6	(8.1)
60	16	(48.5)	39	(55.7)	32	(20.9)	4	(5.4)
80	12	(36.4)	27	(38.6)	12	(7.8)	2	(2.7)
100	6	(19.2)	12	(17.1)	3	(2.0)	0	(0.0)
120	3	(9.1)	5	(7.1)	1	(0.7)	0	(0.0)
150	0	(0.0)	1	(1.4)				

**Table 3:** Overall survival of gastric cancer patients with high HSF1 expression according to the diseases stage (HSF1 probe: **Affy ID: 213756_s_at**).

Month (m)	Tumor Stage							
	Stage I		Stage II		Stage III		Stage IV	
	Number of Patients (%)		Number of Patients (%)		Number of Patients (%)		Number of Patients (%)	
0	33	(100.0)	68	(100.0)	153	(100.0)	73	(100.0)
5	31	(93.9)	65	(95.6)	133	(86.9)	56	(76.7)
10	31	(93.9)	61	(89.7)	113	(73.9)	40	(54.8)
20	28	(84.8)	55	(80.9)	82	(53.6)	23	(31.5)
40	23	(69.7)	46	(67.6)	44	(28.8)	8	(11.0)
60	19	(57.6)	36	(52.9)	30	(19.6)	4	(5.5)
80	13	(39.4)	25	(36.8)	12	(7.8)	3	(4.1)
100	8	(27.3)	12	(17.6)	2	(1.3)	0	(0.0)
120	5	(15.2)	5	(7.4)	1	(0.7)	0	(0.0)
150	0	(0.0)	1	(1.5)				

**Table 4:** HSF1 inhibitors in cancer.

Inhibitors	Mechanism	References
Rocaglamide A	Inhibition of HSF1 binding to HSE	[91]
Rohintib	Inhibition of HSF1 binding to HSE	[91]
KRIBB11	Inhibition of HSF1 transcriptional activity	[81,92]
KNK437	Inhibition of HSF1 transcriptional activity	[81]
Ginsenoside Rg3	Inhibition of HSF1 transcriptional activity	[42]
Cantharidin	Inhibition of HSF1 transcriptional activity	[81,98]
Cardenolide CL-43	Inhibition of HSF1 transcriptional activity	[99]
PW3405	Inhibition of HSF1 phosphorylation	[100]
Compound 1	Inhibition of HSF1 phosphorylation	[101]
Quercetin	Inhibition of HSF1 binding to HSE	[102,103,104]
Fisetin	Inhibition of HSF1 binding to HSE	[76]

## Data Availability

Not applicable.
